# Dimethyl 4-(3,4-dimethoxy­phen­yl)-2,6-dimethyl-1,4-dihydro­pyridine-3,5-dicarboxyl­ate

**DOI:** 10.1107/S160053681001679X

**Published:** 2010-05-15

**Authors:** Tara Shahani, Hoong-Kun Fun, B. Palakshi Reddy, V. Vijayakumar, S. Sarveswari

**Affiliations:** a X-ray Crystallography Unit, School of Physics, Universiti Sains Malaysia, 11800 USM, Penang, Malaysia; b Organic Chemistry Division, School of Advanced Sciences, VIT University, Vellore 632 014, India

## Abstract

In the title compound, C_19_H_23_NO_6_, the 1,4-dihydro­pyridine ring is twisted slightly from planarity, with a maximum deviation of 0.101 (1) Å, and adopts a very flattened boat conformation. The dihedral angle formed between the plane through the four C atoms of the 1,4-dihydro­pyridine ring and the benzene ring is 84.67 (7)°. In the crystal structure, inter­molecular N—H⋯O and C—H⋯O hydrogen bonds link the mol­ecules into a three-dimensional network.

## Related literature

For background to the biological activity of 1,4-dihydro­pyridines, see: Gaudio *et al.* (1994 ▶); Bocker & Guengerich (1986 ▶); Gordeev *et al.* (1996 ▶); Sunkel *et al.* (1992 ▶); Vo *et al.* (1995 ▶); Cooper *et al.* (1992 ▶). For the synthesis of Hantzsch pyridines, see: Rathore *et al.* (2009 ▶). For a related structure, see: Shahani *et al.* (2009 ▶). For reference bond-length data, see: Allen *et al.* (1987 ▶). For puckering parameters, see: Cremer & Pople (1975 ▶). For the stability of the temperature controller used for the data collection, see: Cosier & Glazer (1986 ▶).
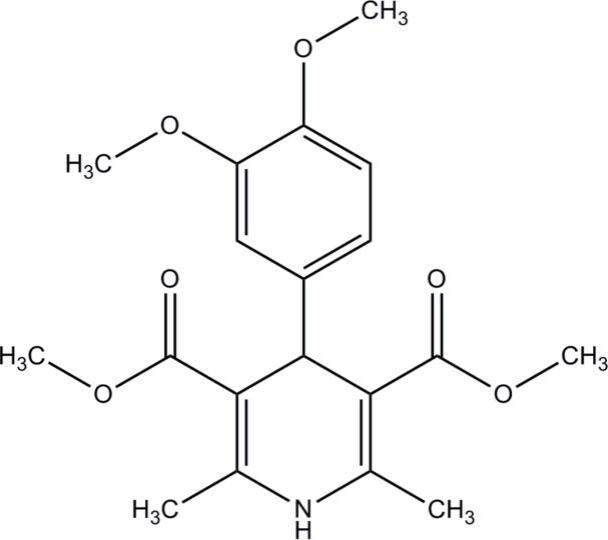


## Experimental

### 

#### Crystal data

C_19_H_23_NO_6_
*M* *_r_* = 361.38
Triclinic, 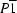

*a* = 7.3883 (6) Å
*b* = 10.0775 (8) Å
*c* = 12.3833 (10) Å
α = 105.372 (2)°
β = 90.255 (2)°
γ = 91.611 (2)°
*V* = 888.60 (12) Å^3^
*Z* = 2
Mo *K*α radiation
μ = 0.10 mm^−1^
*T* = 100 K
0.51 × 0.41 × 0.18 mm

#### Data collection

Bruker APEXII DUO CCD area-detector diffractometer
Absorption correction: multi-scan (*SADABS*; Bruker, 2009 ▶) *T*_min_ = 0.950, *T*_max_ = 0.982
15520 measured reflections
4693 independent reflections
3996 reflections with *I* > 2σ(*I*)
*R*_int_ = 0.023

#### Refinement

*R*[*F* ^2^ > 2σ(*F* ^2^)] = 0.038
*wR*(*F* ^2^) = 0.117
*S* = 1.05
4693 reflections
327 parameters
All H-atom parameters refined
Δρ_max_ = 0.39 e Å^−3^
Δρ_min_ = −0.21 e Å^−3^

### 

Data collection: *APEX2* (Bruker, 2009 ▶); cell refinement: *SAINT* (Bruker, 2009 ▶); data reduction: *SAINT*; program(s) used to solve structure: *SHELXTL* (Sheldrick, 2008 ▶); program(s) used to refine structure: *SHELXTL*; molecular graphics: *SHELXTL*; software used to prepare material for publication: *SHELXTL* and *PLATON* (Spek, 2009 ▶).

## Supplementary Material

Crystal structure: contains datablocks global, I. DOI: 10.1107/S160053681001679X/wn2383sup1.cif

Structure factors: contains datablocks I. DOI: 10.1107/S160053681001679X/wn2383Isup2.hkl

Additional supplementary materials:  crystallographic information; 3D view; checkCIF report

## Figures and Tables

**Table 1:** Hydrogen-bond geometry (Å, °)

*D*—H⋯*A*	*D*—H	H⋯*A*	*D*⋯*A*	*D*—H⋯*A*
N1—H1*N*1⋯O5^i^	0.88 (2)	2.21 (2)	3.0750 (13)	166.7 (16)
C12—H12*C*⋯O4^ii^	0.926 (16)	2.550 (17)	3.4120 (17)	155.0 (13)
C15—H15*C*⋯O2^iii^	0.968 (19)	2.501 (19)	3.4136 (17)	157.0 (17)
C17—H17*C*⋯O5^i^	0.98 (2)	2.52 (2)	3.4318 (14)	154.9 (15)
C19—H19*A*⋯O5^iv^	0.954 (17)	2.562 (18)	3.5023 (15)	168.7 (13)

Symmetry codes: (i) 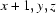; (ii) 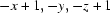; (iii) 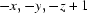; (iv) 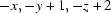.

**Fig. 1.**
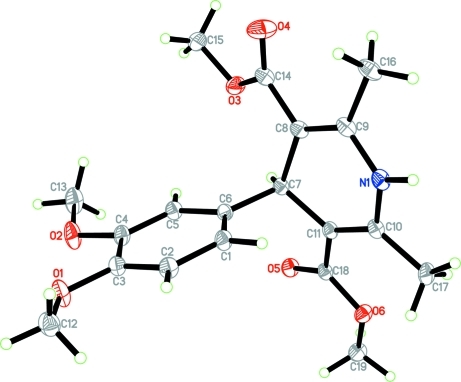
The molecular structure of the title compound, showing 50% probability displacement ellipsoids and the atom numbering scheme. Hydrogen atoms are shown as spheres of arbitrary radius.

**Fig. 2.**
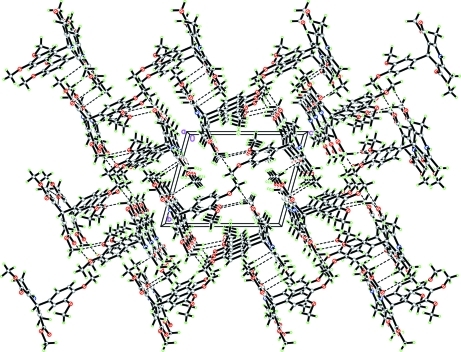
The crystal packing of the title compound, viewed along the a axis. Intermolecular hydrogen bonds are shown as dashed lines.

## supplementary crystallographic information

### Comment

Hantzsch 1,4-dihydropyridines (1,4-DHPs) are biologically active compounds which include various vasodilator, antihypertensive, bronchodilator, heptaprotective, antitumor, antimutagenic, geroprotective and antidiabetic agents (Gaudio *et al.*, 1994) Nifedipine, nitrendipine, nimodipine *etc.* have found commercial utility as calcium channel blockers (Bocker & Guengerich, 1986; Gordeev *et al.*, 1996). For the treatment of congestive heart failure, a number of DHP calcium antagonists have been introduced (Sunkel *et al.*, 1992; Vo *et al.*, 1995). Some DHPs have been introduced as neuroprotectant and cognition enhancers. In addition, a number of DHPs with platelet antiaggregatory activity have also been discovered (Cooper *et al.*, 1992).

In the title compound (Fig. 1), the 1,4-dihydropyridine (C7–C9/N1/C10/C11) ring is slightly twisted from planarity, with a maximum deviation of 0.101 (1) Å at atom C11, and adopts a very flattened boat conformation (Cremer & Pople, 1975), with puckering parameters Q = 0.2412 (12) Å, Θ = 75.8 (3)° & φ = 183.4 (3)°. The dihedral angle formed between the plane through the four atoms C8-C11 of the 1,4-dihydropyridine and benzene (C1–C6) ring is 84.67 (7)°. The bond lengths (Allen *et al.*, 1987) and angles are within normal ranges and comparable to those in a closely related crystal structure (Shahani *et al.*, 2009).

In the crystal packing (Fig. 2), intermolecular N1—H1N1···O5, C12—H12C···O4, C15—H15C···O2, C17—H17C···O5 and C19—H19A···O5 hydrogen bonds (Table 1) link the molecules into a three-dimensional network.

### Experimental

Dimethyl-1,4-dihydro-2,6-dimethyl-4-(3,4-dimethoxyphenyl)-3,5-pyridine dicarboxylate was prepared according to the Hantzsch pyridine synthesis (Rathore *et al.*, 2009). A mixture of 3,4-dimethoxybenzaldehyde (10 mmol), methyl acetoacetate (20 mmol) and ammonium acetate (10 mmol) was heated at 80 °C for 2 hours (monitored by TLC). After completion of the reaction, the mixture was cooled to room temperature and allowed to stand for 1 day to obtain a solid product. This solid was washed with diethyl ether and the title compound obtained from the washings by evaporation. The purity of the crude product was checked by TLC and recrystallized using acetone and diethyl ether. *Mp*: 156-158 °C, IR (KBr):ν (cm^-1^), 3361, 2994, 1701, 1654, 1217.

### Refinement

All H atoms were located in a difference map and were refined freely. [N–H = 0.879 (19) Å, C–H = 0.93 (2)–0.986 (16) Å].

### Crystal data

**Table:**

C_19_H_23_NO_6_	*Z* = 2
*M**_r_* = 361.38	*F*(000) = 384
Triclinic, *P*1	*D*_x_ = 1.351 Mg m^−^^3^
Hall symbol: -P 1	Mo *K*α radiation, λ = 0.71073 Å
*a* = 7.3883 (6) Å	Cell parameters from 8704 reflections
*b* = 10.0775 (8) Å	θ = 2.3–30.1°
*c* = 12.3833 (10) Å	µ = 0.10 mm^−^^1^
α = 105.372 (2)°	*T* = 100 K
β = 90.255 (2)°	Block, colourless
γ = 91.611 (2)°	0.51 × 0.41 × 0.18 mm
*V* = 888.60 (12) Å^3^	

### Data collection

**Table:**

Bruker APEXII DUO CCD area-detector diffractometer	4693 independent reflections
Radiation source: fine-focus sealed tube	3996 reflections with *I* > 2σ(*I*)
graphite	*R*_int_ = 0.023
φ and ω scans	θ_max_ = 29.0°, θ_min_ = 1.7°
Absorption correction: multi-scan (*SADABS*; Bruker, 2009)	*h* = −10→9
*T*_min_ = 0.950, *T*_max_ = 0.982	*k* = −13→13
15520 measured reflections	*l* = −16→16

### Refinement

**Table:**

Refinement on *F*^2^	Primary atom site location: structure-invariant direct methods
Least-squares matrix: full	Secondary atom site location: difference Fourier map
*R*[*F*^2^ > 2σ(*F*^2^)] = 0.038	Hydrogen site location: inferred from neighbouring sites
*wR*(*F*^2^) = 0.117	All H-atom parameters refined
*S* = 1.05	*w* = 1/[σ^2^(*F*_o_^2^) + (0.0633*P*)^2^ + 0.3175*P*] where *P* = (*F*_o_^2^ + 2*F*_c_^2^)/3
4693 reflections	(Δ/σ)_max_ < 0.001
327 parameters	Δρ_max_ = 0.39 e Å^−^^3^
0 restraints	Δρ_min_ = −0.21 e Å^−^^3^

### Special details

**Table:**

Experimental. The crystal was placed in the cold stream of an Oxford Cyrosystems Cobra open-flow nitrogen cryostat (Cosier & Glazer, 1986) operating at 100.0 (1)K.
Geometry. All esds (except the esd in the dihedral angle between two l.s. planes) are estimated using the full covariance matrix. The cell esds are taken into account individually in the estimation of esds in distances, angles and torsion angles; correlations between esds in cell parameters are only used when they are defined by crystal symmetry. An approximate (isotropic) treatment of cell esds is used for estimating esds involving l.s. planes.
Refinement. Refinement of *F*^2^ against ALL reflections. The weighted *R*-factor wR and goodness of fit *S* are based on *F*^2^, conventional *R*-factors *R* are based on *F*, with *F* set to zero for negative *F*^2^. The threshold expression of *F*^2^ > σ(*F*^2^) is used only for calculating *R*-factors(gt) etc. and is not relevant to the choice of reflections for refinement. *R*-factors based on *F*^2^ are statistically about twice as large as those based on *F*, and *R*- factors based on ALL data will be even larger.

### Fractional atomic coordinates and isotropic or equivalent isotropic displacement parameters (Å^2^)

**Table:**

	*x*	*y*	*z*	*U*_iso_*/*U*_eq_	
O1	0.13177 (12)	0.35766 (10)	0.50655 (8)	0.0253 (2)	
O2	−0.09815 (12)	0.19240 (10)	0.56457 (8)	0.0271 (2)	
O3	0.21291 (12)	−0.12807 (9)	0.77077 (7)	0.02175 (19)	
O4	0.49285 (13)	−0.19568 (10)	0.71696 (9)	0.0311 (2)	
O5	0.05882 (11)	0.30006 (9)	1.00996 (7)	0.01900 (18)	
O6	0.26916 (11)	0.44327 (8)	1.11470 (7)	0.01862 (18)	
N1	0.67178 (13)	0.18380 (10)	0.94785 (8)	0.0175 (2)	
C1	0.39341 (15)	0.27760 (12)	0.73593 (10)	0.0175 (2)	
C2	0.35435 (16)	0.33318 (12)	0.64648 (10)	0.0189 (2)	
C3	0.18797 (16)	0.30518 (12)	0.59181 (9)	0.0178 (2)	
C4	0.06117 (15)	0.21698 (12)	0.62489 (10)	0.0179 (2)	
C5	0.10199 (15)	0.16280 (11)	0.71366 (9)	0.0166 (2)	
C6	0.26780 (14)	0.19386 (11)	0.77130 (9)	0.0147 (2)	
C7	0.30862 (14)	0.13710 (11)	0.87152 (9)	0.0141 (2)	
C8	0.45041 (15)	0.02726 (11)	0.84314 (9)	0.0161 (2)	
C9	0.62445 (15)	0.05553 (12)	0.87803 (10)	0.0172 (2)	
C10	0.54594 (15)	0.27558 (11)	1.00303 (9)	0.0156 (2)	
C11	0.36775 (14)	0.25185 (11)	0.97297 (9)	0.0146 (2)	
C12	0.26124 (19)	0.43765 (14)	0.46362 (11)	0.0240 (3)	
C13	−0.21854 (18)	0.08780 (16)	0.58366 (12)	0.0281 (3)	
C14	0.39486 (16)	−0.10892 (12)	0.77100 (10)	0.0188 (2)	
C15	0.14106 (19)	−0.25176 (13)	0.69309 (11)	0.0254 (3)	
C16	0.77848 (16)	−0.04195 (13)	0.85144 (11)	0.0223 (2)	
C17	0.62731 (15)	0.39180 (13)	1.09351 (10)	0.0195 (2)	
C18	0.21850 (15)	0.33172 (11)	1.03232 (9)	0.0147 (2)	
C19	0.12427 (16)	0.52520 (12)	1.17271 (10)	0.0201 (2)	
H1A	0.509 (2)	0.3021 (17)	0.7758 (14)	0.027 (4)*	
H2A	0.446 (2)	0.3860 (16)	0.6208 (14)	0.025 (4)*	
H5A	0.015 (2)	0.1061 (15)	0.7381 (13)	0.019 (4)*	
H7A	0.195 (2)	0.0945 (15)	0.8880 (13)	0.019 (4)*	
H12A	0.193 (3)	0.4694 (18)	0.4082 (16)	0.036 (5)*	
H12B	0.306 (2)	0.5163 (17)	0.5209 (14)	0.027 (4)*	
H12C	0.357 (2)	0.3847 (17)	0.4310 (14)	0.026 (4)*	
H13A	−0.160 (2)	0.0001 (18)	0.5738 (14)	0.027 (4)*	
H13B	−0.272 (2)	0.1130 (16)	0.6595 (14)	0.023 (4)*	
H13C	−0.316 (3)	0.0809 (19)	0.5306 (16)	0.040 (5)*	
H15A	0.196 (2)	−0.3330 (18)	0.7040 (15)	0.030 (4)*	
H15B	0.009 (3)	−0.2556 (18)	0.7063 (15)	0.038 (5)*	
H15C	0.163 (3)	−0.2468 (18)	0.6172 (16)	0.034 (4)*	
H16A	0.887 (3)	−0.001 (2)	0.8987 (17)	0.044 (5)*	
H16B	0.812 (3)	−0.0635 (19)	0.7690 (16)	0.038 (5)*	
H16C	0.746 (3)	−0.128 (2)	0.8611 (17)	0.044 (5)*	
H17A	0.594 (2)	0.4817 (16)	1.0829 (13)	0.025 (4)*	
H17B	0.576 (2)	0.3880 (17)	1.1657 (15)	0.032 (4)*	
H17C	0.760 (3)	0.3858 (18)	1.0926 (15)	0.034 (4)*	
H19A	0.058 (2)	0.5670 (17)	1.1248 (15)	0.031 (4)*	
H19B	0.046 (3)	0.4709 (18)	1.2075 (15)	0.034 (4)*	
H19C	0.187 (2)	0.5943 (18)	1.2325 (15)	0.032 (4)*	
H1N1	0.786 (3)	0.2040 (17)	0.9681 (15)	0.031 (4)*	

### Atomic displacement parameters (Å^2^)

**Table:**

	*U*^11^	*U*^22^	*U*^33^	*U*^12^	*U*^13^	*U*^23^
O1	0.0210 (4)	0.0347 (5)	0.0258 (4)	−0.0061 (4)	−0.0070 (3)	0.0186 (4)
O2	0.0165 (4)	0.0412 (5)	0.0284 (5)	−0.0104 (4)	−0.0108 (3)	0.0188 (4)
O3	0.0172 (4)	0.0185 (4)	0.0264 (4)	−0.0006 (3)	−0.0042 (3)	0.0006 (3)
O4	0.0259 (5)	0.0216 (4)	0.0420 (6)	0.0043 (4)	0.0084 (4)	0.0011 (4)
O5	0.0101 (4)	0.0214 (4)	0.0246 (4)	−0.0004 (3)	−0.0016 (3)	0.0046 (3)
O6	0.0120 (4)	0.0197 (4)	0.0216 (4)	−0.0002 (3)	−0.0008 (3)	0.0012 (3)
N1	0.0081 (4)	0.0219 (5)	0.0234 (5)	−0.0006 (3)	−0.0009 (3)	0.0078 (4)
C1	0.0122 (5)	0.0209 (5)	0.0197 (5)	−0.0025 (4)	−0.0032 (4)	0.0064 (4)
C2	0.0162 (5)	0.0207 (5)	0.0208 (5)	−0.0040 (4)	−0.0011 (4)	0.0077 (4)
C3	0.0173 (5)	0.0199 (5)	0.0167 (5)	−0.0002 (4)	−0.0020 (4)	0.0063 (4)
C4	0.0123 (5)	0.0223 (5)	0.0190 (5)	−0.0021 (4)	−0.0038 (4)	0.0058 (4)
C5	0.0123 (5)	0.0191 (5)	0.0189 (5)	−0.0023 (4)	−0.0011 (4)	0.0059 (4)
C6	0.0121 (5)	0.0155 (5)	0.0162 (5)	0.0010 (4)	−0.0010 (4)	0.0037 (4)
C7	0.0094 (5)	0.0159 (5)	0.0177 (5)	−0.0008 (4)	−0.0012 (4)	0.0056 (4)
C8	0.0131 (5)	0.0166 (5)	0.0199 (5)	0.0010 (4)	0.0014 (4)	0.0070 (4)
C9	0.0136 (5)	0.0197 (5)	0.0210 (5)	0.0019 (4)	0.0028 (4)	0.0099 (4)
C10	0.0118 (5)	0.0192 (5)	0.0178 (5)	−0.0008 (4)	0.0001 (4)	0.0084 (4)
C11	0.0114 (5)	0.0170 (5)	0.0164 (5)	−0.0007 (4)	−0.0010 (4)	0.0064 (4)
C12	0.0246 (6)	0.0260 (6)	0.0240 (6)	−0.0037 (5)	−0.0018 (5)	0.0119 (5)
C13	0.0171 (6)	0.0402 (8)	0.0290 (7)	−0.0107 (5)	−0.0077 (5)	0.0141 (6)
C14	0.0189 (5)	0.0177 (5)	0.0216 (5)	0.0018 (4)	0.0008 (4)	0.0083 (4)
C15	0.0271 (7)	0.0188 (6)	0.0275 (6)	−0.0011 (5)	−0.0094 (5)	0.0017 (5)
C16	0.0141 (5)	0.0243 (6)	0.0320 (6)	0.0050 (4)	0.0037 (4)	0.0132 (5)
C17	0.0106 (5)	0.0248 (6)	0.0229 (5)	−0.0032 (4)	−0.0039 (4)	0.0064 (4)
C18	0.0126 (5)	0.0163 (5)	0.0165 (5)	−0.0008 (4)	−0.0010 (4)	0.0070 (4)
C19	0.0152 (5)	0.0214 (5)	0.0218 (5)	0.0019 (4)	0.0011 (4)	0.0021 (4)

### Geometric parameters (Å, °)

**Table:**

O1—C3	1.3679 (14)	C7—H7A	0.980 (16)
O1—C12	1.4268 (15)	C8—C9	1.3530 (16)
O2—C4	1.3721 (13)	C8—C14	1.4713 (16)
O2—C13	1.4282 (15)	C9—C16	1.5044 (16)
O3—C14	1.3526 (15)	C10—C11	1.3637 (15)
O3—C15	1.4434 (14)	C10—C17	1.4991 (16)
O4—C14	1.2108 (15)	C11—C18	1.4654 (15)
O5—C18	1.2245 (14)	C12—H12A	0.974 (19)
O6—C18	1.3452 (13)	C12—H12B	0.962 (17)
O6—C19	1.4442 (14)	C12—H12C	0.927 (18)
N1—C10	1.3805 (14)	C13—H13A	0.972 (17)
N1—C9	1.3856 (15)	C13—H13B	0.992 (16)
N1—H1N1	0.879 (19)	C13—H13C	0.96 (2)
C1—C6	1.3860 (15)	C15—H15A	0.963 (18)
C1—C2	1.3997 (16)	C15—H15B	0.99 (2)
C1—H1A	0.977 (18)	C15—H15C	0.968 (19)
C2—C3	1.3860 (16)	C16—H16A	1.01 (2)
C2—H2A	0.956 (17)	C16—H16B	1.018 (19)
C3—C4	1.4090 (16)	C16—H16C	0.93 (2)
C4—C5	1.3862 (15)	C17—H17A	0.986 (16)
C5—C6	1.4002 (15)	C17—H17B	0.983 (18)
C5—H5A	0.952 (16)	C17—H17C	0.983 (19)
C6—C7	1.5300 (15)	C19—H19A	0.955 (18)
C7—C11	1.5176 (15)	C19—H19B	0.964 (19)
C7—C8	1.5194 (15)	C19—H19C	0.977 (19)
			
C3—O1—C12	117.10 (10)	O1—C12—H12A	104.1 (11)
C4—O2—C13	117.19 (9)	O1—C12—H12B	111.7 (10)
C14—O3—C15	115.84 (10)	H12A—C12—H12B	109.0 (14)
C18—O6—C19	116.00 (9)	O1—C12—H12C	111.0 (10)
C10—N1—C9	123.01 (10)	H12A—C12—H12C	111.1 (15)
C10—N1—H1N1	116.4 (12)	H12B—C12—H12C	109.8 (15)
C9—N1—H1N1	119.8 (11)	O2—C13—H13A	112.5 (10)
C6—C1—C2	121.00 (10)	O2—C13—H13B	112.9 (9)
C6—C1—H1A	120.0 (10)	H13A—C13—H13B	107.8 (13)
C2—C1—H1A	118.9 (10)	O2—C13—H13C	105.0 (11)
C3—C2—C1	120.15 (10)	H13A—C13—H13C	111.4 (15)
C3—C2—H2A	120.4 (10)	H13B—C13—H13C	107.3 (15)
C1—C2—H2A	119.3 (10)	O4—C14—O3	122.11 (11)
O1—C3—C2	125.46 (10)	O4—C14—C8	126.90 (11)
O1—C3—C4	115.17 (10)	O3—C14—C8	110.99 (10)
C2—C3—C4	119.36 (10)	O3—C15—H15A	111.8 (11)
O2—C4—C5	124.82 (10)	O3—C15—H15B	106.8 (11)
O2—C4—C3	115.45 (10)	H15A—C15—H15B	109.8 (15)
C5—C4—C3	119.73 (10)	O3—C15—H15C	109.4 (11)
C4—C5—C6	121.17 (10)	H15A—C15—H15C	109.0 (15)
C4—C5—H5A	119.9 (9)	H15B—C15—H15C	109.9 (16)
C6—C5—H5A	118.9 (9)	C9—C16—H16A	109.7 (11)
C1—C6—C5	118.54 (10)	C9—C16—H16B	111.5 (11)
C1—C6—C7	120.84 (9)	H16A—C16—H16B	109.3 (16)
C5—C6—C7	120.61 (9)	C9—C16—H16C	111.6 (12)
C11—C7—C8	110.65 (9)	H16A—C16—H16C	111.3 (16)
C11—C7—C6	111.02 (8)	H16B—C16—H16C	103.2 (16)
C8—C7—C6	111.31 (9)	C10—C17—H17A	111.1 (10)
C11—C7—H7A	109.2 (9)	C10—C17—H17B	108.8 (10)
C8—C7—H7A	108.6 (9)	H17A—C17—H17B	105.9 (14)
C6—C7—H7A	105.9 (9)	C10—C17—H17C	109.5 (10)
C9—C8—C14	120.87 (10)	H17A—C17—H17C	109.0 (14)
C9—C8—C7	121.20 (10)	H17B—C17—H17C	112.5 (15)
C14—C8—C7	117.85 (9)	O5—C18—O6	121.78 (10)
C8—C9—N1	119.50 (10)	O5—C18—C11	123.15 (10)
C8—C9—C16	126.29 (11)	O6—C18—C11	115.07 (9)
N1—C9—C16	114.19 (10)	O6—C19—H19A	112.1 (11)
C11—C10—N1	119.03 (10)	O6—C19—H19B	110.5 (11)
C11—C10—C17	127.59 (10)	H19A—C19—H19B	111.5 (16)
N1—C10—C17	113.37 (9)	O6—C19—H19C	103.4 (11)
C10—C11—C18	124.66 (10)	H19A—C19—H19C	111.4 (15)
C10—C11—C7	121.19 (10)	H19B—C19—H19C	107.6 (15)
C18—C11—C7	114.15 (9)		
			
C6—C1—C2—C3	0.14 (18)	C14—C8—C9—C16	−0.70 (18)
C12—O1—C3—C2	6.09 (18)	C7—C8—C9—C16	−177.18 (10)
C12—O1—C3—C4	−174.62 (11)	C10—N1—C9—C8	13.87 (16)
C1—C2—C3—O1	177.31 (11)	C10—N1—C9—C16	−164.32 (10)
C1—C2—C3—C4	−1.96 (18)	C9—N1—C10—C11	−12.47 (16)
C13—O2—C4—C5	−9.85 (18)	C9—N1—C10—C17	166.41 (10)
C13—O2—C4—C3	170.68 (11)	N1—C10—C11—C18	172.89 (10)
O1—C3—C4—O2	2.12 (16)	C17—C10—C11—C18	−5.82 (18)
C2—C3—C4—O2	−178.53 (11)	N1—C10—C11—C7	−7.50 (16)
O1—C3—C4—C5	−177.37 (10)	C17—C10—C11—C7	173.78 (10)
C2—C3—C4—C5	1.97 (17)	C8—C7—C11—C10	22.90 (14)
O2—C4—C5—C6	−179.60 (11)	C6—C7—C11—C10	−101.21 (12)
C3—C4—C5—C6	−0.16 (18)	C8—C7—C11—C18	−157.45 (9)
C2—C1—C6—C5	1.66 (17)	C6—C7—C11—C18	78.44 (11)
C2—C1—C6—C7	−178.00 (10)	C15—O3—C14—O4	−6.40 (17)
C4—C5—C6—C1	−1.65 (17)	C15—O3—C14—C8	173.30 (10)
C4—C5—C6—C7	178.01 (10)	C9—C8—C14—O4	−16.87 (19)
C1—C6—C7—C11	52.50 (13)	C7—C8—C14—O4	159.72 (12)
C5—C6—C7—C11	−127.15 (11)	C9—C8—C14—O3	163.45 (10)
C1—C6—C7—C8	−71.23 (13)	C7—C8—C14—O3	−19.96 (14)
C5—C6—C7—C8	109.12 (11)	C19—O6—C18—O5	−1.71 (15)
C11—C7—C8—C9	−21.56 (14)	C19—O6—C18—C11	178.71 (9)
C6—C7—C8—C9	102.39 (12)	C10—C11—C18—O5	−172.80 (11)
C11—C7—C8—C14	161.86 (9)	C7—C11—C18—O5	7.57 (15)
C6—C7—C8—C14	−74.20 (12)	C10—C11—C18—O6	6.78 (16)
C14—C8—C9—N1	−178.65 (10)	C7—C11—C18—O6	−172.85 (9)
C7—C8—C9—N1	4.87 (16)		

### Hydrogen-bond geometry (Å, °)

**Table:**

*D*—H···*A*	*D*—H	H···*A*	*D*···*A*	*D*—H···*A*
N1—H1N1···O5^i^	0.88 (2)	2.21 (2)	3.0750 (13)	166.7 (16)
C12—H12C···O4^ii^	0.926 (16)	2.550 (17)	3.4120 (17)	155.0 (13)
C15—H15C···O2^iii^	0.968 (19)	2.501 (19)	3.4136 (17)	157.0 (17)
C17—H17C···O5^i^	0.98 (2)	2.52 (2)	3.4318 (14)	154.9 (15)
C19—H19A···O5^iv^	0.954 (17)	2.562 (18)	3.5023 (15)	168.7 (13)

Symmetry codes: (i) *x*+1, *y*, *z*; (ii) −*x*+1, −*y*, −*z*+1; (iii) −*x*, −*y*, −*z*+1; (iv) −*x*, −*y*+1, −*z*+2.
